# The effects of exopolysaccharides and exopolysaccharide-producing *Lactobacillus* on the intestinal microbiome of zebrafish (*Danio rerio*)

**DOI:** 10.1186/s12866-020-01990-6

**Published:** 2020-10-06

**Authors:** Chenchen Ma, Hongyang Guo, Haibo Chang, Shi Huang, Shuaiming Jiang, Dongxue Huo, Jiachao Zhang, Xiaopeng Zhu

**Affiliations:** 1 College of Food Science and Engineering, School of Life and Pharmaceutical Sciences, Hainan University, Haikou, 570228 Hainan P. R. China; 2 Key Laboratory of Tropical Biological Resources, Ministry of Education, Hainan University, Haikou, 570228 Hainan P. R. China; 3 Single-Cell Center, Qingdao Institute of Bioenergy and Bioprocess Technology, Chinese Academy of Sciences, Qingdao, 266101 Shandong People’s Republic of China

**Keywords:** Exopolysaccharides, Intestinal microbiota, Zebrafish, *Lactobacillus*, Intestinal inflammation, Short-chain fatty acid

## Abstract

**Background:**

Numerous studies have reported the health-promoting effects of exopolysaccharides (EPSs) in in vitro models; however, a functional evaluation of EPSs will provide additional knowledge of EPS-microbe interactions by in vivo intestinal microbial model. In the present study, high-throughput amplicon sequencing, short-chain fatty acid (SCFAs) and intestinal inflammation evaluation were performed to explore the potential benefits of exopolysaccharides (EPSs) and EPS-producing *Lactobacillus* (HNUB20 group) using the healthy zebrafish (*Danio rerio*) model.

**Results:**

The results based on microbial taxonomic analysis revealed that the abundance of four genera, *Ochrobactrum, Sediminibacterium, Sphingomonas* and *Sphingobium*, were increased in the control group in comparison to HNUB20 group. *Pelomonas* spp. levels were significantly higher and that of the genera *Lactobacillus* and *Brachybacterium* were significantly decreased in EPS group compared with control group. PICRUSt based functional prediction of gut microbiota metabolic pathways indicated that significantly lower abundance was found for transcription, and membrane transport, whereas folding, sorting and degradation and energy metabolism had significantly higher abundance after HNUB20 treatment. Two metabolic pathways, including metabolism and endocrine functions, were more abundant in the EPS group than control group. Similar to the HNUB20 group, transcription was also decreased in the EPS group compared with the control group. However, SCFAs and immune indexes indicated EPS and HNUB20 performed limited efficacy in the healthy zebrafish.

**Conclusions:**

The present intestinal microbial model-based study indicated that EPSs and high-yield EPS-producing *Lactobacillus* can shake the structure of intestinal microbiota, but cannot change SCFAs presence and intestinal inflammation.

## Background

*Lactobacillus* and *Bifidobacterium spp*, have been widely used as probiotics to prevent disease and improve host health via the gut microbiota [1, 2]. Exopolysaccharides (EPSs) are carbohydrate polymers composed of monosaccharides, including homopolysaccharides and heteropolysaccharides, and are secreted by many bacteria [3, 4]. In particular, beneficial health effects of EPSs produced by lactic acid bacteria (LABs) have been focused [5]. The human gut is inhabited by a large number of bacteria, which are involved in maintenance of host homeostasis. As a potential prebiotic, EPSs are also a source of energy and nutrients available to the gut microbiota [6]. *Lactobacillus plantarum* and *Bifidobacterium* derived EPSs can increase the content of short-chain fatty acids (SCFAs) in faeces [7, 8]. Previous study reported that SCFAs are produced by gut microbiota as products of dietary fiber fermentation and may support host antibody responses [9]. EPS from *Lactobacillus plantarum* NCU116 can regulate intestinal epithelial barrier function [10]. Furthermore, EPS of *Lactobacillus paraplantarum* BGCG11 reduces inflammatory hyperalgesia in rats [11]. In addition, an anti-obesity effect in mice [12], promotion of probiotic bacteria growth [13] and a cholesterol-lowering effect in mice [14] have also been reported. Notably, EPSs also enhanced the growth of *Clostridium* in vitro [15]. Nonetheless, it remains unclear whether EPSs are actually beneficial to the host. Thus, in vivo studies are urgently needed to explore the interaction among EPSs, EPS-producing strains and the gut microbiota to confirm or refute the capability of LAB EPSs to exert a healthy effect on the host.

In recent decades, use of zebrafish (*Danio rerio*) as a model system to study the correlation of gut microbiota and host health with bacterial exposure has expanded due to the high conservation of genes between zebrafish and humans, with 87% conservation overall [16]. Their intestines are small, facilitating the study of the entire intestinal microbiota [17, 18]. Given the highly conserved nature of innate defense in zebrafish, it represents an ideal model system for inflammatory bowel disease [19]. An EPS-producing bacteria have shown positive health properties in zebrafish models [20]. A previous study has shown that *Lactobacillus* spp. may colonize the intestinal tract of zebrafish [21]. Nevertheless, evaluation of the health benefits of *Lactobacillus* EPS and EPS-producing *Lactobacillus* in a zebrafish adult model has remained unexplored.

Thus, to further understand the effect of EPS and EPS-producing strains in the healthy zebrafish, we performed preliminary research investigating the gut microbiota, microbiota metabolic pathways, SCFAs presence and intestinal inflammation. The results of this study provide novel data on health benefits of *Lactobacilus* and *Lactobacillus* derived EPS, and we highlight the fact that probiotics and prebiotics need more evaluation in vivo using animal models.

## Results

### Bacterial community richness and diversity in healthy zebrafish

Chao1 and ACE indices represent the community richness, and Shannon and Simpson indices represent community diversity. We compared ACE, Chao1, Shannon, and Simpson indices in day seven. EPS compared to control group, there were no significant differences (*P* values > 0.05, by Wilcoxon rank sum test) in all indices. Chao1 index in HNUB20 group was lower than that in the control group, while no significant differences were found in other indices (Table 1).

**Table 1:** Comparison of alpha diversities among three groups

	HNUB20	Con	EPS	***P*** value	***P*** value
	(mean ± SD)	(mean ± SD)	(mean ± SD)	(B20 vs Con)	(EPS vs Con)
Simpson	0.86 ± 0.04	0.91 ± 0.03	0.88 ± 0.01	0.095	0.31
Chao1	660.14 ± 178.33	701.20 ± 182.64	647.12 ± 137.88	0.008	0.548
ACE	680.19 ± 201.47	711.82 ± 165.30	652.21 ± 146.32	0.814	0.691
Shannon	4.66 ± 0.57	5.46 ± 0.69	4.96 ± 0.13	0.222	0.548

### Bacterial community composition and comparison of different genera

**Fig. 1:**
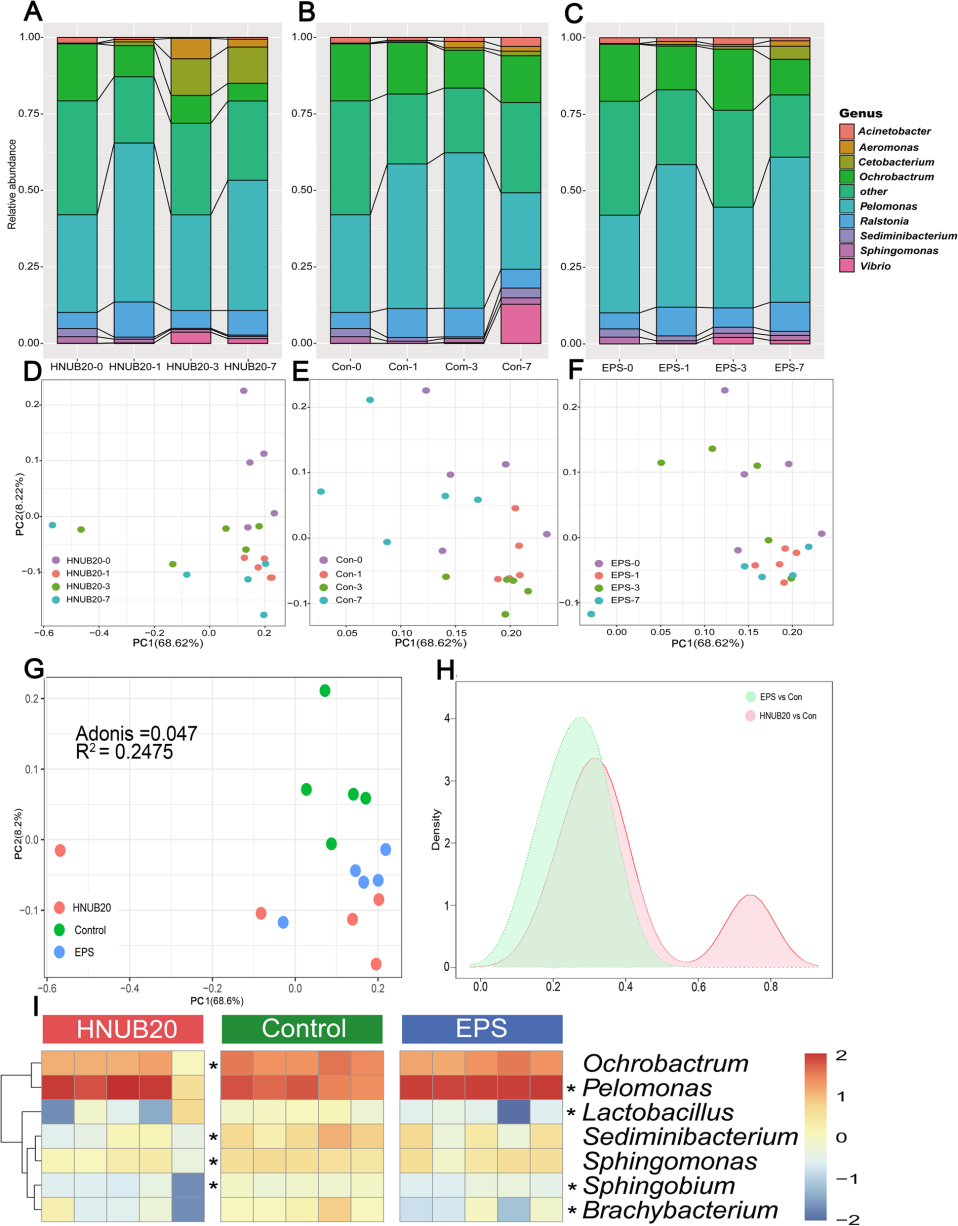
Bacterial community composition and comparison of different genera. The histogram shows changes in the core microbiota at different time points (**a-c**). Principal coordinate analysis (PCoA) reveals the microbial structure based on weighted UniFrac distances in the healthy zebrafish at different time points (**d-f**), seventh days of PCOA could be found in (**g**). The density plot shows the degree of change after EPS and *Lactobacillus fermentum* HNUB20 treatment (Fig. H1). The different genera are illustrated with a heatmap, and the star represents different genera compared with the control group (**i**) using the Wilcoxon rank sum test, *P* < 0.01)

The core genera were defined as those present in carried at least by 80% of samples, and with average relative abundance at 1% [22]. Changes in the core microbiota, including Proteobacteria (*Acinetobacter, Aeromonas, Ochrobactrum, Pelomonas, Ralstonia, Sphingomonas and Vibrio*), *Fusobacteria* (Cetobacterium), Bacteroidetes (*Sediminibacterium*) of the zebrafish intestine are presented as histograms in Fig. 1a-c. *Pelomonas* and *Ralstonia* maintained the same variation trend (increase, decrease, increase) during 7 days of HNUB20 and EPS treatment, as did *Cetobacterium* (increase, increase, decrease). We compared the intestinal structure based on PCoA of weighted UniFrac distances (Fig. 1d-f), and the results revealed dynamic changes in the microbiota over time among the three groups. We found the intestinal microbiota of the EPS and HNUB20 groups were most stable compared with the control group. The abundance of bacteria in phyla level and core genera at 7 days could be found in more detail (see Additional file 6 and Additional file 7).

To analyse the beta diversity of different groups, Weighted UniFrac distances and Adonis analysis were performed for day 7 samples. The results showed that the differences in microbial structure among the three groups were significant and distinct, with a *P* value of 0.047, a R value of 0.2475 (Fig. 1g). Furthermore, the structural similarity of the intestinal microbiota between the EPS and control groups and the *L. fermentum* HNUB20 and control groups were compared, and the results are shown in Fig. 1h. The similarity between the EPS and the control groups was higher than that between the *L. fermentum* HNUB20 and control groups, indicating that EPS had a lesser effect on the intestinal microbial community than did strain HNUB20.

Next, different intestinal microbiota genera (relative abundance > 1%, *P* < 0.01 by the Wilcoxon rank sum test) were compared, as shown by a heatmap in Fig. 1i, in which the stars represent different genera. Four different genera, *Ochrobactrum*, *Sediminibacterium*, *Sphingomonas* and *Sphingobium*, showed significantly higher abundance in the control group versus the HNUB20-treated group. In addition, *Pelomonas* was present at significantly higher abundance in the EPS group than in the control group, and *Lactobacillus* and *Brachybacterium* were in higher abundance in the control group than in the EPS group.

### Comparison of microbial metabolic pathways predicted by PICRUSt

**Fig. 2:**
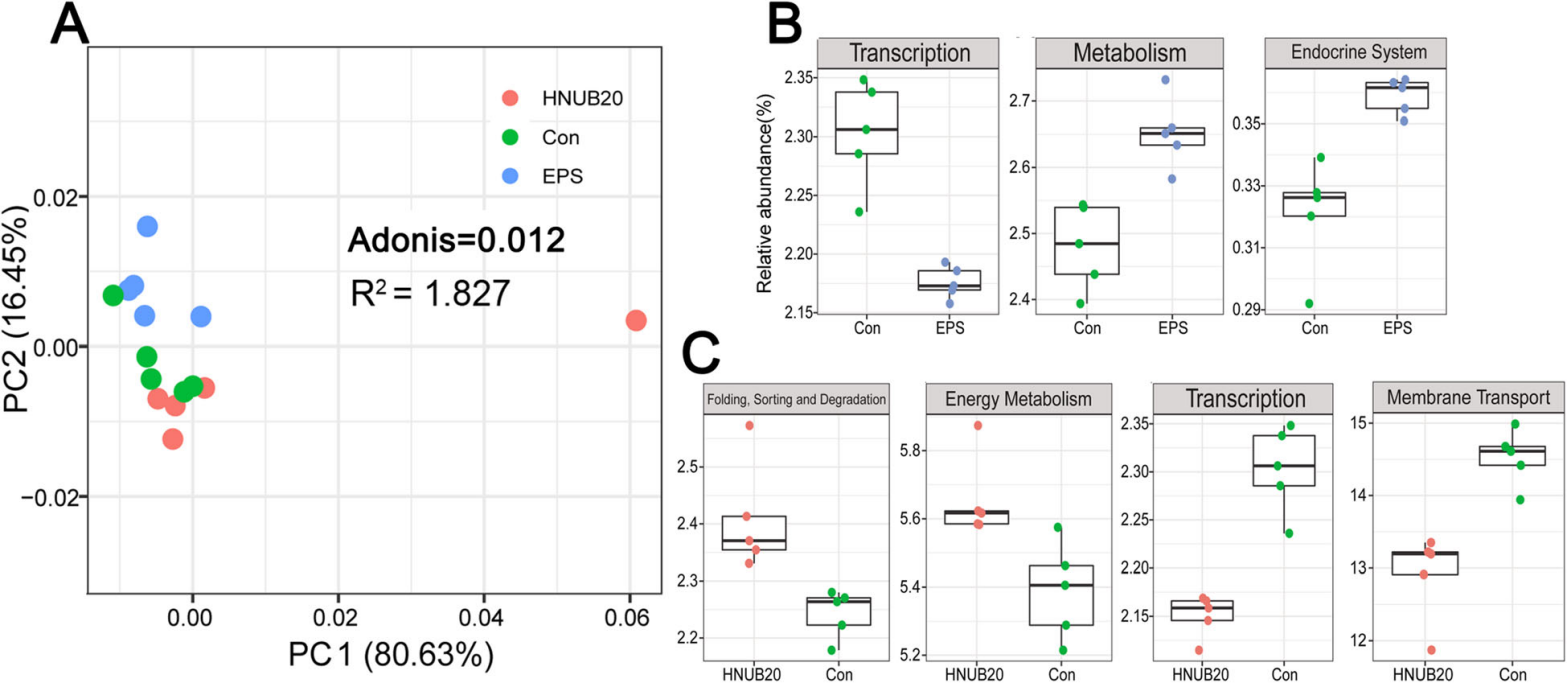
Principal component analysis (PCA) of metabolic pathways and different metabolic pathways in level 2. Adonis analysis was conducted to permute *P* value by 999 permutations (**a**). The Wilcoxon rank sum test was used and considered significant at *P* < 0.01 (**b**)

To better understand differences in microbial functions among the three groups, functional features were predicted by PICRUSt for day 7 samples. The abundance of metabolic pathways in level 2 based on KEGG database could be found in more detail (see Additional file 8). First, we performed PCA analysis based on metabolic pathway relative abundance in level 2; second, Adonis analysis was performed to calculate differences among the three groups. The clusters with a *P* value of 0.012, a R value of 1.827 are shown in Fig. 2a, and the different metabolic pathways are shown (relative abundance > 1%, *P* < 0.01, by the Wilcoxon rank sum test) using a boxplot (Fig. 2b-c). Compared with the control group, the HNUB20 group exhibited five differentially enriched metabolic pathways: significantly lower abundance was found for transcription, and membrane transport, whereas folding, sorting and degradation and energy metabolism had significantly higher abundance. Two metabolic pathways, including metabolism and endocrine functions, were more abundant in the EPS group than in the control group. Similar to the HNUB20 group, transcription was also decreased in the EPS group compared with the control group. Therefore, the functional structure was changed by consuming EPS and *L. fermentum* HNUB20 for 7 days.

### The impacts of EPS and HNUB20 on the intestinal SCFAs

**Fig. 3:**
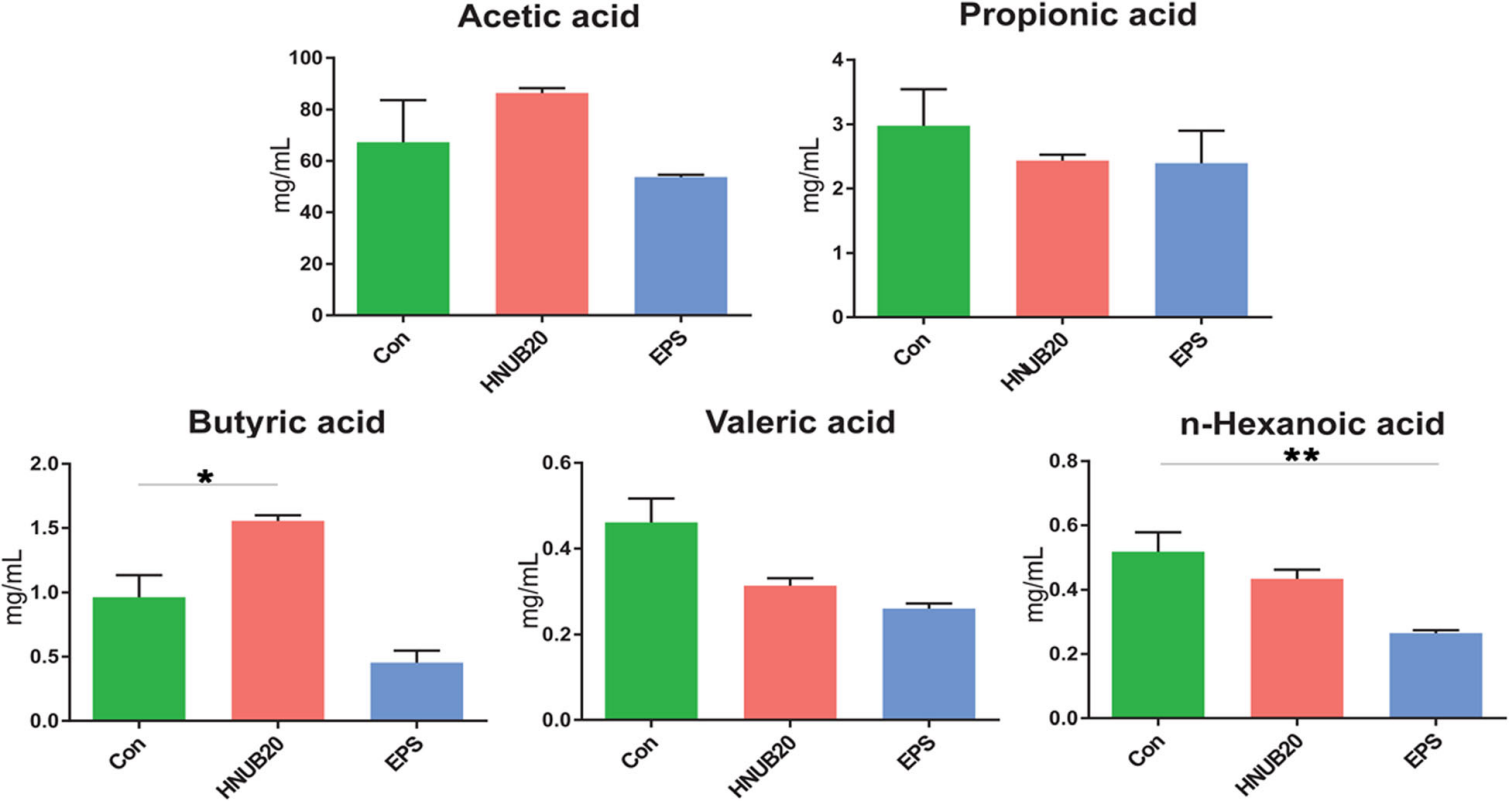
Responses to SCFAs. The statistical analyses were performed by T-test (two-tailed). *: *p* < 0.05 **: *p* < 0.01

We detected SCFAs of the whole zebrafish intestine, including acetic acid, propionic acid, butyric acid, valeric acid and n-hexanoic acid. In the healthy zebrafish (Fig. 3), the abundance of acetic acid and propionic acid showed no significant difference by EPS and HNUB20 treatment. Compared with control group, n-hexanoic acid were significantly higher than EPS group. An increase in butyric acid was observed by HNUB20 treatment. The results indicated EPS and HNUB20 had limited influence on SCFAs abudance of the whole zebrafish intestines. Here, although, we did not accurately describe the content of SCFAs produced by the intestinal microbiota. Our PICRUSt results showed that there was no difference in the citrate cycle (TCA cycle) among the three groups (see Additional file 4), and there may be no difference in the consumption of SCFAs. Therefore, it was feasible to determine the SCFA of the whole intestine according to previous research [23].

### Immune indexes response to EPS and EPS producing lactobacillus comsumption

**Fig. 4:**
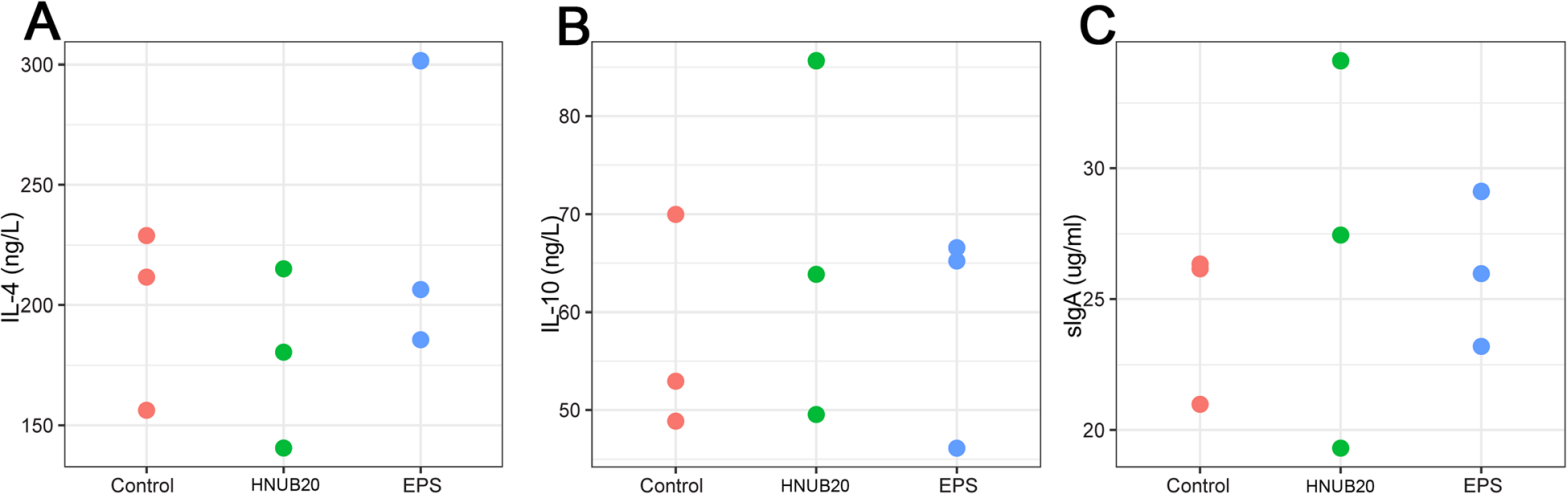
Responses to immune indexes. The statistical analyses were performed by T-test (two-tailed). *: *p* < 0.05 **: *p* < 0.01

Key inflammatory mediators or immunoglobulin IL-4, IL-10, sIgA were detected by ELISA, no significant differences were observed in the healthy zebrafish among all indexes (Fig. 4).

### The correlation network revealed the potential modulation process of EPS and *Lactobacillus fermentum* HNUB20 in the healthy zebrafish

**Fig. 5:**
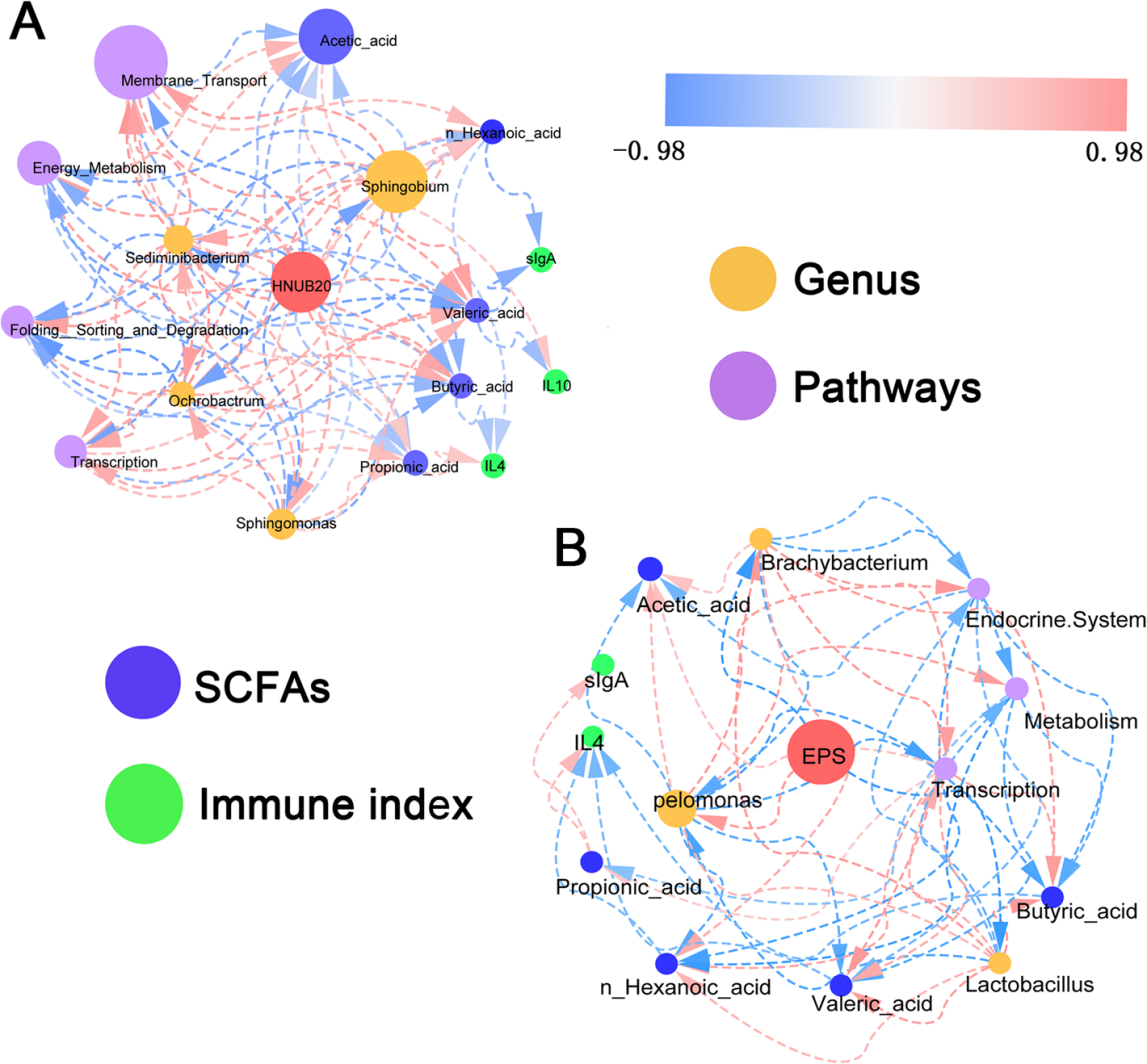
Correlation network analysis among EPS, *Lactobacillus fermentum* HNUB20, different genera, metabolic pathways, SCFAs and immune indexes. Correlations were estimated by Spearman rank correlation coefficient. Absolute correlations below 0.4 were masked to show all exclusively significant signals. The colour (red, positive; blue, negative) are proportional to the correlations. The node size of genera and pathways is proportional to the mean abundance in the respective cohorts

Differentially abundant genera and metabolic pathways were revealed by the above analysis in the healthy zebrafish. To better show correlations between the differentially abundant genera and metabolic pathways, Spearman’s correlation coefficient was calculated based on the relative abundance of different genera, SCFAs, immune indexes and metabolic pathways. We then performed two network analyses to explore the dynamic processes occurring when zebrafish consume EPS and *L. fermentum* HNUB20 (Fig. 5). The network showed high correlations among strain HNUB20, differentially abundant genera, SCFAs, immune indexes and metabolic pathways. It could be observed the intake of HNUB20 or EPS affected intestinal microbiota and metabolic profiles. Meanwhile SCFAs stimulated the host’s immune response, accompanied with the rise or fall of immune indexes including sIgA, IL-4 and IL-10. Although limited effects were found by EPS and EPS-producing strain in this study, the potential correlation network may show that the EPS and HNUB20-drived changes in intestinal microbiota and the gut metabolites were involved in the bioactivity performance of EPS and HNUB20. This may be one of the potential mechanisms of EPS and HNUB20 regulation of immune immunity.

## Discussion

In this study, we found EPSs and HNUB20 can shake the structure of intestinal microbiota, while the effects on specific genera were obviously different. The specific manifestation is that there is no same differential genus. Although the secretion of EPS is a physiological characteristic of HNUB20, from its point of view of intestinal microbiota, we should evaluate a strain more comprehensively, rather than just one characteristic. Then, commonalities between EPS and HNUB20 were found that is not strong enough to stimulate SCFAs and immunity. We think that the reason is that the regulation of specific intestinal microbes, including SCFAs-secreting bacteria is still insufficient.

Gut microbiota composition may be important an indicator of host health [24, 25]. In our study, no significant differences in alpha diversity were found among the three groups of healthy zebrafish. An earlier study showed a sex dependent effect on alpha diversity, whereby alpha diversity was significantly different among males, with no difference among females [26]. Thus, sex may affect changes in alpha diversity. Our research included females and males that were not evaluated separately. It has been reported that EPS from *Lactobacillus buchneri* TCP016 had no effect on mouse alpha diversity of microbiota [27]. In general, larger sample sizes may be needed to confirm the factors affecting alpha diversity. Regardless, the core microbiota showed changes after EPS and *L. fermentum* HNUB20 treatment. Nine genera were defined as the core microbiota with average relative abundances greater than 1%. Three genera, *Aeromonas*, *Vibrio* and *Cetobacterium*, are considered to belong to core intestinal microbiota in domesticated and caught zebrafish [28]. *Vibrio* and *Cetobacterium* are also known as core microbiota of adult zebrafish [29], which is consistent with our study. *L. fermentum* HNUB20 inhibited the growth of pathogenic bacteria (*Ochrobactrum*) [30, 31], and bacteria associated with disease biomarkers (*Sediminibacterium*, *Sphingomonas*, *Sphingobium*) [32–34]. HNUB20 derived EPS can decrease the level of *Pelomonas*, which is a biomarker of bladder cancer [32]. It also inhibited the growth of *Brachybacterium*, which is an end-stage renal disease biomarker (*Brachybacterium*) [35].

Most related studies to date have been performed in vitro. However, additional in vivo research is needed to evaluate the health benefits and properties of EPSs and EPS-producing strains. One of the first questions is whether EPSs contribute to the colonization of strains. We detected the abundance of *Lactobacillus fermentum* by qPCR based on specific primers of *Lactobacillus fermentum* [36], and the results can be found in more detail (see Additional file 2). However, no significant differences were found. In fact, adhesion is one of the properties frequently assessed in the search for potential probiotic strains [37]. Several studies have been performed with Caco-2 cells in vitro, showing that EPS can help other bacteria strains to adhere to these cells [20]. On the other hand, another study showed that non-EPS-producing strains persist longer in mice [38] and that the presence of EPS surrounding the bacterial surface might reduce adherence [39]. Nonetheless, strain adherence to human GI cells in vitro may be a poor indicator of in vivo colonization due to a myriad of host and microbiome factors that are absent in the in vitro setting [40]. In addition, EPS may not be a good complete carbon source for *Lactobacillus*. Most studies have reported increases in *Bifidobacterium* abundance by EPS treatment [41, 42]. EPS may have two sides (promote or inhibit colonization) for EPS-producing *Lactobacillus* to the host and the microbiota.

The SCFAs were considered as the key bacterial metabolites, which can modulate immune response in gut by controlling the expression of genes involved in synthesis of molecules necessary for plasma B cell differentiation [43]. A study by Rio-Covian et al. revealed that EPS promotes the release of SCFAs [44], while limited effect also was found with EPS treatment [45]. Propionic acid and butyric acid contribute to an anti-inflammatory by induce apoptosis of neutrophils [46]. Butyric acid also exerts immunomodulatory effect by effecting on intestinal macrophages result in a reduction in the production of pro-inflammatory cytokines [47]. However, based on our data, no consistent responses were found on SCFAs with EPS and HNUB20 treatment in the healthy zebrafish, though HNUB20 can enhance intestinal immune function and promote intestinal health through butyrate production. IgA is the most abundant immunoglobulin subtype present in epithelial mucus, which captures antigens and prevents them from binding to cell surface receptors. Dendritic cells (DCs) operate on mesenteric lymph nodes by sampling of polysaccharide and bacteria, which is responsible for regulatory T (Treg) to produce IL-10, and IgA^+^ B cell (responsible for IgA^+^ plasma cell to produce IgA) [48]. EPS extracted from *L. mesenteroides* strain NTM048 (ranging in size from 10 to 40 kDa) possesses the ability to increase IgA levels [49]. In addition, EPS from *Lactobacillus casei* WXD030 (average molecular weight of 37,370 Da) promoted IL-4 and IL-10 expression [50]. However, a limited response to EPS of strain HNUB20 (EPS was estimated to be 45,065 Da) was found in this study in the healthy zebrafish. Some previous studies have focused on large or high molecular weight EPSs, which appear to act as suppressors of the immune response, which may explain the difference in responses observed [51] in the healthy zebrafish. Collectively, we speculated that the HNUB20 may act as a transient microbe that does not permanently colonize the gut yet still shake the composition of the host intestinal microbiota and have a limited impact on SCFAs presence and intestinal inflammation of the host.

## Conclusions

In conclusion, the present study described the dynamic profile of the response of the zebrafish intestinal microbiota, SCFAs and immune indexes to the HNUB20 and EPS intakes. The present intestinal microbial model-based study indicated that EPSs and high-yield EPS-producing *Lactobacillus* can shake the structure of intestinal microbiota, but cannot change SCFAs presence and intestinal inflammation.

## Methods

### Screening of EPS-producing lactic acid bacteria and preparation of crude EPS

In our previous studies, we isolated and identified 178 lactic acid bacteria from fermented seafoods of the Hainan area, China [52]. These lactic acid bacteria were screened for EPS production. The methods of Ai et al. [53] were adopted and improved. Cultures were inoculated at 2% (v/v) and cultured in MRS medium for approximately 48 h at 37 °C. Trichloroacetic acid (TCA) with a final concentration of 3% was added, and then placed in the refrigerator for 4 hours to precipitate proteins [54]. The clear supernatant was collected after centrifugation at 12,000 g for 15 min, and EPS was precipitated by adding three volumes of cold ethanol followed by overnight storage at 4 °C. After centrifugation (12,000×g for 20 min), EPS was resuspended in ddH_2_O, dialyzed against running water for 48 h (8000–14,000 Da), and collected by centrifugation at 12,000×g for 30 min. Pelleted EPS was resuspended in ddH_2_O. To determine the yield of EPS, the phenol-sulfuric acid method was used with glucose as a standard [55] (y = 0.0179x-0.0302 R^2^ = 0.9967). *Lactobacillus fermentum* HNUB20 was found to produce the most EPS, at approximately 98.8 mg/L (the screening results can be found in supplemental material [see Additional file 5]). The crude EPS of strain HNUB20 was prepared from 10 L culture by ethanol precipitation, followed by dialysis and freeze drying. Isolated EPS was stored at 4 °C for further experiments. The HPLC analysis of EPS is presented (see Additional file 1). Based on the results, the molecular weight of EPS is 45,065 Da.

### Animal experimental design, diet preparation and sample collection

Adult zebrafish (including male and female adults, 3 months old, average mass 500 ± 50 mg, average body length 3.2 ± 0.1 cm) used in the experiment were obtained from a local supplier in Haikou and cultured at the aquatic experimental animal facility of the College of Food Science and Technology, Hainan University. The zebrafish were acclimated to laboratory conditions for 14 days in aerated dechlorinated tap water prior to experiments. According to the standard zebrafish breeding protocol, the acclimation and subsequent treatment period were conducted at 28 ± 0.5 °C, with a light/dark cycle of 14 h/10 h. The water was replaced at 9 a.m. every day to avoid the growth of pathogenic bacteria. The fish were fed commercial feed (Sanyou Chuangmei, China) daily in the amount of 3% of the total zebrafish weight. After 14 days of acclimation, zebrafish were randomly distributed into three groups (Control, EPS and HNUB20 Group), with three 3-L tanks for each group (24 cm × 13 cm × 12 cm) and 16 fish in each tank. The EPS group zebrafish were fed the control diet plus 1% (m/m) crude EPS. *L. fermentum* HNUB20 cells were pelleted by centrifugation and resuspended in 50 μL of saline, which was mixed with feed at 8.0 log_10_ CFU/g diet.

Zebrafish were anesthetized by immersion in 0.2 mg/ml tricaine (MS-222) [56] and then placed on an ice plate for dissection sampling. All experiments were carried out in accordance with the National Institutes of Health Guide for the Care and Use of Laboratory Animals (NIH Publications No.8023). All of the experimental and animal care procedures were approved by the Ethics Committee of Hainan University (Permit Number: HNU-EC-20180701). Fifteen intestinal samples of the healthy zebrafish were collected at four points during the experiment (baseline, first day, third days, and seventh days). The whole intestines (esophagus to anus) from three animals were mixed into one sample, giving a totally five pooled samples for further microbiota analysis. The flow chart can be found in more detail [see Additional file 3].

### DNA extraction

500 μL of TE (10 Tris-EDTA, pH 8.0 Tris-HCl 0.1 mol/L) was added to homogenize the samples. Samples were stored at − 20 °C until analysis. Metagenomic DNA was extracted from the zebrafish intestinal samples using a CWBIO Stool Genomic DNA Kit (CW2092, CWBIO, China). We assessed the quality of the metagenomic DNA by 0.8% agarose gel electrophoresis before sequencing. Isolated metagenomic DNA was stored at − 20 °C and used as the template for further analysis.

### DNA sequencing

The Shanghai Personal Biotechnology company amplified the DNA coding for the V3-V4 region of the 16S ribosomal RNA (rRNA) gene, as described previously [57]. We added a set of 6-nucleotide barcodes to the universal forward primer 338F (5′-ACTCCTACGGGAGGCAGCA-3′) and reverse primer 806R (5′-GGACTACHVGGGTWTCTAAT-3′). Quantification of PCR products was performed using an Agilent DNA 1000 Kit and Agilent 2100 Bioanalyzer (Agilent Technologies, USA) according to the manufacturer’s instructions. The amplification products were pooled in equimolar ratios at a final concentration of 100 nmol/L each and sequenced using the Illumina MiSeq platform with the barcoded primers.

### SCFA analysis

Short fatty acids were analyzed by a gas chromatography-mass spectrometry (GC-MS) as previously described [58]. In short, the whole gut tissue samples (esophagus to anus) of the healthy zebrafish from five animals were weighted, followed by addition of 2 mL H_2_SO_4_ (0.5 mol/L) and extracted 30 min by ultrasonic vibration (40 °C, 35 kHz). Then, 1 mL of diethyl ether was added, samples were stored at 4 °C for 5 min and centrifuged (12,000 g for 5 min). Finally, 100 μL of acetone was added for GC-MS after diethyl ether was removed. An Agilent 7890A-5975C GC-MS and Agilent DB-WAX (0.25 mm × 0.25 μm × 50 m) columns were used. The GC conditions were as follows: inlet temperature: 250 °C; carrier gas flow rate: 1.5 mL/min; shunt ratio 3:1 and injection amount of sample was 1 μL. The GC temperature program was as follows: 70 °C hold for 3.0 min, increase to 200 °C by 10 °C/min, 200 °C hold for 2.0 min, increased to 180 °C by 8 °C/min, 180 °C hold for 10.0 min, and increased to 250 °C for 10 min by 15 °C/min. The MS conditions were as follows: the ion source chamber was set at 230 °C with the transfer line temperature set to 250 °C, the electron energy was 70 eV, and the full scan was 35 to 550 Da. All samples were tested three times and the average value was used as the result.

### Enzyme-linked immunosorbent assays (ELISA)

The whole gut tissue (esophagus to anus) of all group from two zebrafish were mixed into one sample, weighed, homogenized in 7.2 μL/mg PBS (pH 6.0, 50 mM) and centrifuged (13,000 g for 5 min), giving totally three pooled samples for further analysis, all samples were tested three times and the average value was used as the result. Supernatants were used for interleukin-4 (IL-4), interleukin-10 (IL-10), and secretory IgA (sIgA) quantification using ELISA kits, described in the manual instructions (X-Y Biotechnology, Shanghai, CN). Absorption at 450 nm was determined using a microplate reader (SpectraMax M2, MD).

### Bioinformatics and statistical analyses

Low-quality sequences from 16S rDNA sequencing results were removed after trimming based on the original raw data. FLASH and QIIME (v1.8.0) USEARCH software were used for further sequence quality analysis [59]. Representative OTUs (operational taxonomic unit) were selected and annotated using the Greengenes database [60]. PICRUSt (Phylogenetic Communities by Reconstruction of Unobserved States) was applied to predict the functional features of the zebrafish intestinal microbiota based on the OTU table [61]. Statistical analyses were conducted using R software. The data were corrected for multiple testing, and the *p*-value was the adjusted p-value by the “p.adjust” command in R. Differences in the abundance of genera and metabolic pathways were assessed by Wilcoxon rank sum test and considered significant at *P* < 0.01. Only those pathways and genera with more than 0.1% average abundance and only genera present in at least 20% of the samples were used for statistical analysis by Wilcoxon rank sum test. Graph drawing and Principal component analysis (PCA) were performed using the ggplot2 package [62]. Principal coordinate analysis (PCoA) of weighted Unifrac distance was performed in R using the ade4 package [63]. Adonis analysis was conducted using the vegan package, and the permuted *P* value was obtained by 999 permutations. Heatmaps, which were employed to show the presence of different bacterial genera were generated using the pheatmap package. Correlations between microbes among groups were calculated using the Spearman rank correlation (psych package) coefficient and visualized as a network in Cytoscape (v 3.4) [64]. T-test (two-tailed) was used to compare the difference for cytokines and SCFAs.

## Supplementary information

**Additional file 1.** The HPLC analysis of EPS.

**Additional file 2 **The qPCR results of *Lactobacillus fermentum.*

**Additional file 3.** The workflow of the study.

**Additional file 4.** The relative abundance of citrate cycle (TCA cycle) among the three groups.

**Additional file 5.** The screening results.

**Additional file 6.** The abundance of bacteria at phyla level.

**Additional file 7.** The abundance of core genera.

**Additional file 8.** The abundance of metabolic pathways in level 2.

## Data Availability

The datasets generated during the current study are available in the [NCBI] repository, [https://www.ncbi.nlm.nih.gov/bioproject/PRJNA530096].

## Abbreviations

EPSs: Exopolysaccharides
LAB: Lactic acid bacteria
PCoA: Principal coordinate analysis
PICRUSt: Phylogenetic Communities by Reconstruction of Unobserved States
