# Supplementary figures and images for: The effects of exopolysaccharides and exopolysaccharide-producing Lactobacillus on the intestinal microbiome of zebrafish (Danio rerio)
Source: BMC Microbiol. 2020 Oct 6;20:300. doi: 10.1186/s12866-020-01990-6 (PMC7539446; doi:10.1186/s12866-020-01990-6)

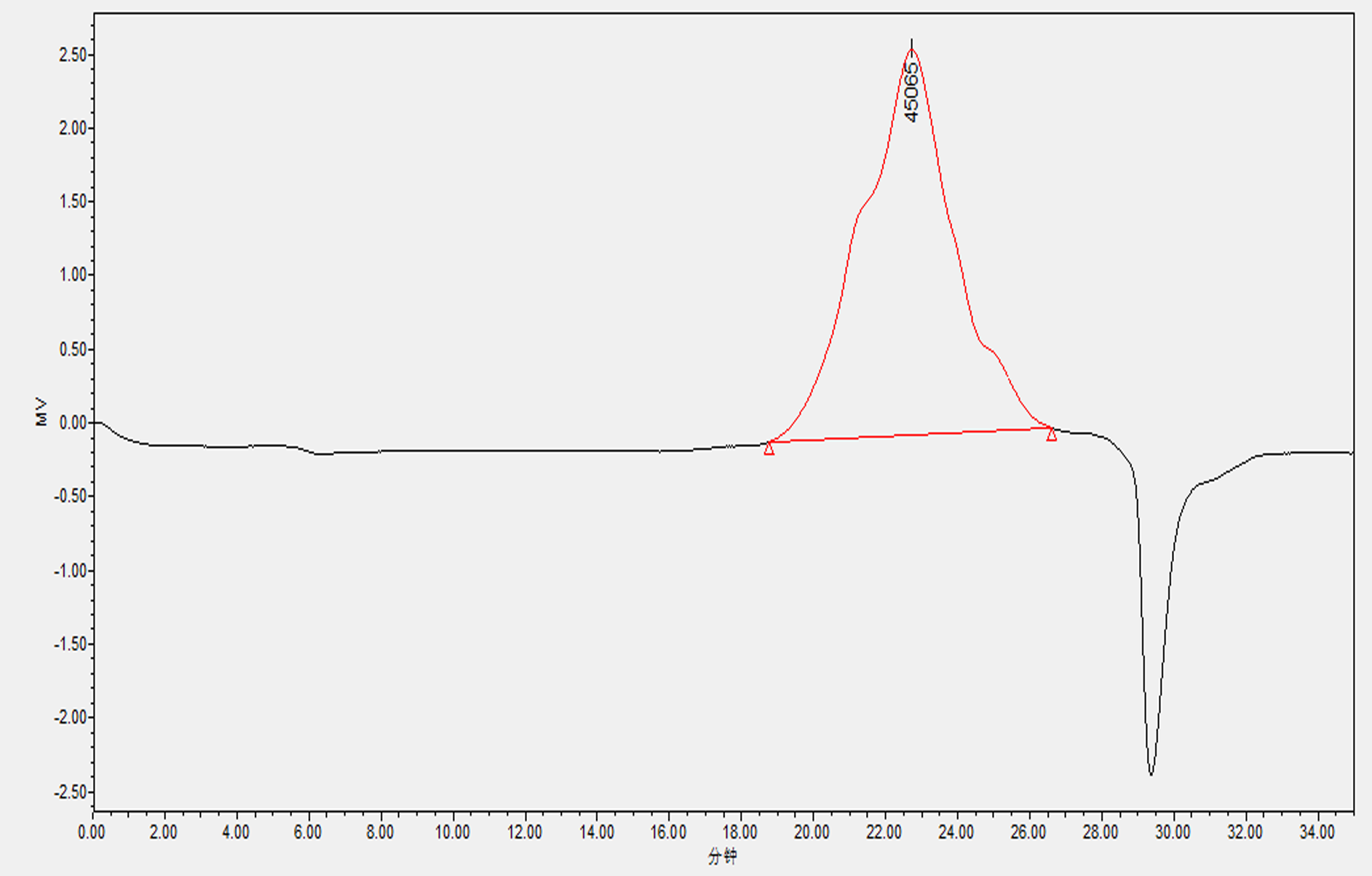

Supplement: Supplementary file 1 — Additional file 1. The HPLC analysis of EPS. [file 12866_2020_1990_MOESM1_ESM.tif]

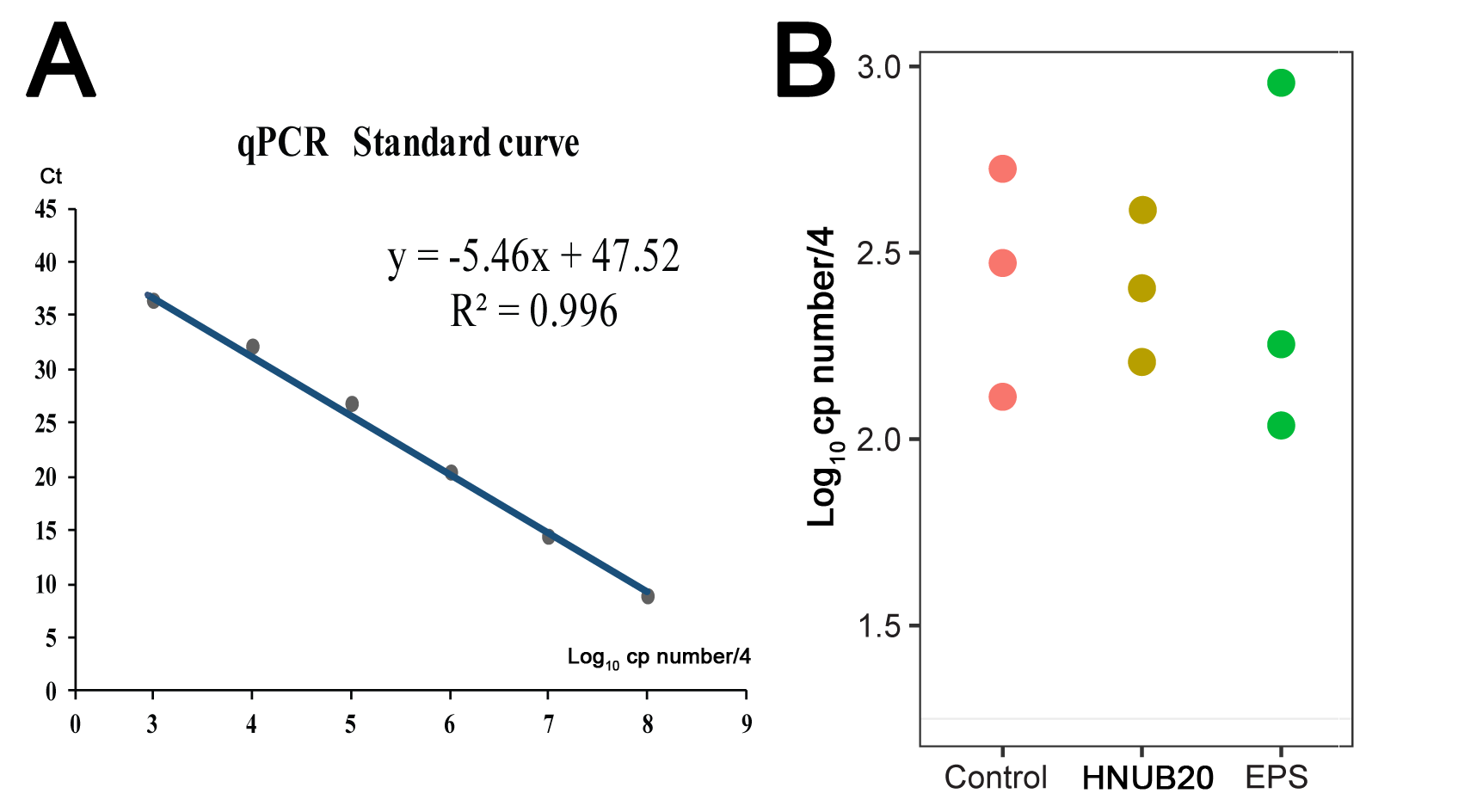

Supplement: Supplementary file 2 — Additional file 2 The qPCR results of Lactobacillus fermentum. [file 12866_2020_1990_MOESM2_ESM.tif]

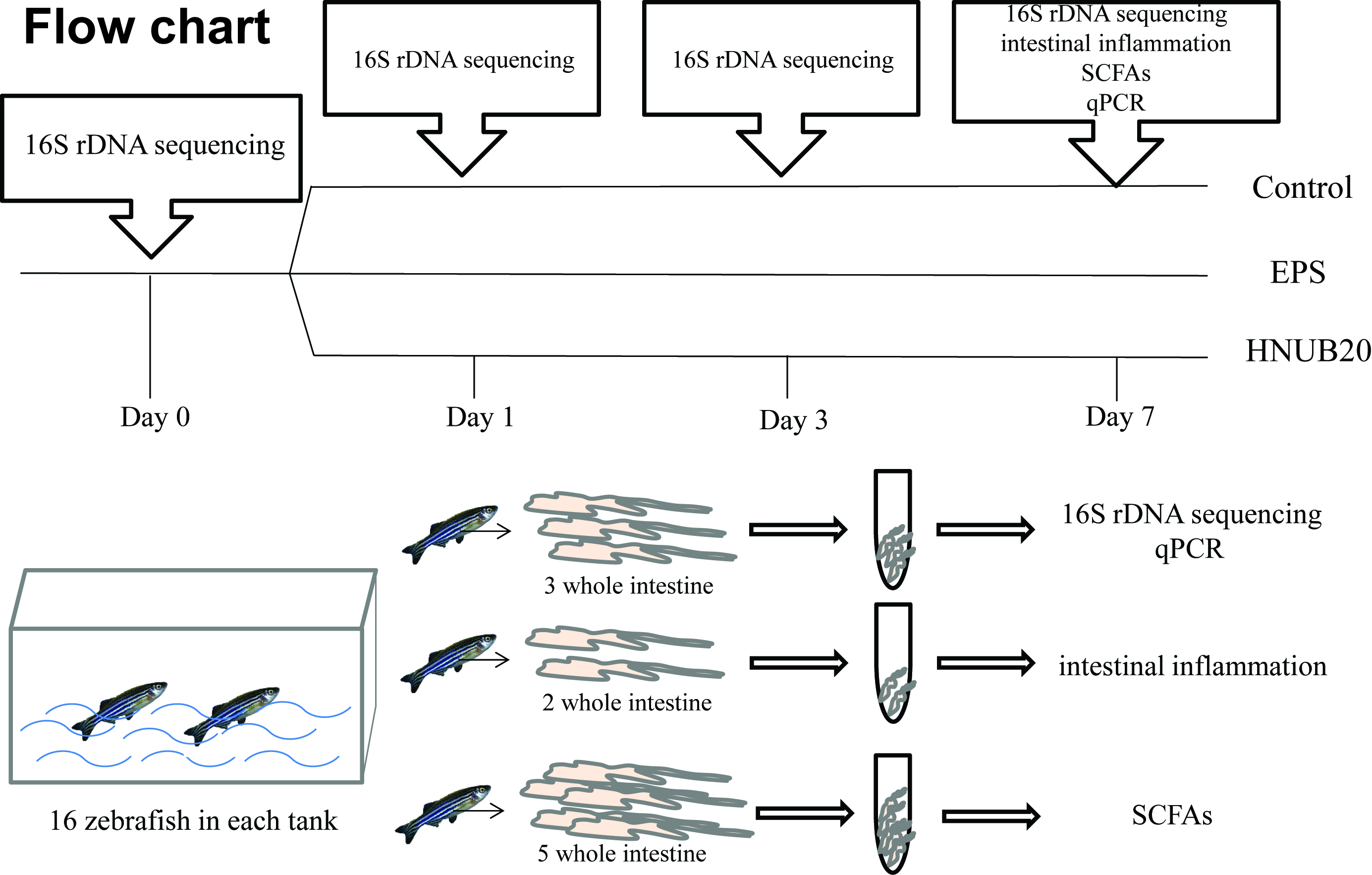

Supplement: Supplementary file 3 — Additional file 3. The workflow of the study. [file 12866_2020_1990_MOESM3_ESM.tif]

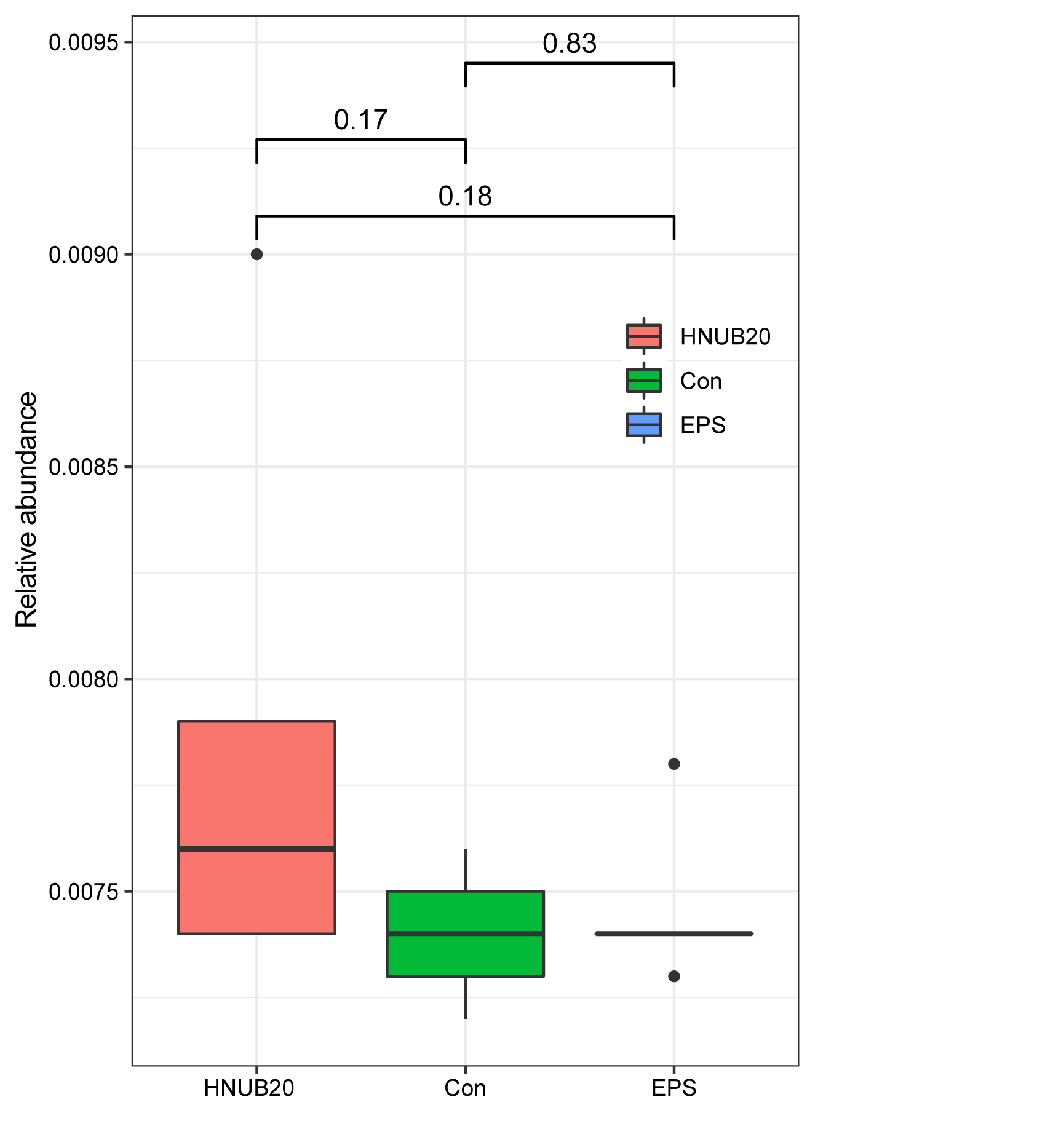

Supplement: Supplementary file 4 — Additional file 4. The relative abundance of citrate cycle (TCA cycle) among the three groups. [file 12866_2020_1990_MOESM4_ESM.tif]
